# Short post-injection seizure duration is associated with reduced power of ictal brain perfusion SPECT to lateralize the seizure onset zone

**DOI:** 10.1186/s13550-024-01095-5

**Published:** 2024-04-17

**Authors:** Amir Karimzadeh, Kian Baradaran-Salimi, Berthold Voges, Ivayla Apostolova, Thomas Sauvigny, Michael Lanz, Susanne Klutmann, Stefan Stodieck, Philipp T. Meyer, Ralph Buchert

**Affiliations:** 1 Department of Diagnostic and Interventional Radiology and Nuclear Medicine, University Medical Center Hamburg-Eppendorf, Martinistr. 52, 20246 Hamburg, Germany; 2 Department of Neurology and Epileptology, Protestant Hospital Alsterdorf, Hamburg, Germany; 3 Department of Neurosurgery, University Medical Center Hamburg-Eppendorf, Hamburg, Germany; 4 Department of Nuclear Medicine, Medical Center-University of Freiburg, Faculty of Medicine, University of Freiburg, Freiburg, Germany

**Keywords:** Epilepsy, Perfusion, SPECT, Seizure duration, Injection latency, Cerebral blood flow, Technetium Tc 99 m exametazime, Technetium Tc 99 m bicisate

## Abstract

**Background:**

The aim of this study was to assess the impact of the post-injection electrical seizure duration on the identification of the seizure onset zone (SOZ) in ictal brain perfusion SPECT in presurgical evaluation of drug-resistant epilepsy.

**Methods:**

176 ictal SPECT performed with ^99m^Tc-HMPAO (n = 140) or -ECD (n = 36) were included retrospectively. Visual interpretation of the SPECT images (together with individual MRI and statistical hyperperfusion maps) with respect to lateralization (right, left, none) and localization (temporal, frontal, parietal, occipital) of the SOZ was performed by 3 independent readers. Between-readers agreement was characterized by Fleiss’ κ. An ictal SPECT was considered "lateralizing" if all readers agreed on right or left hemisphere. It was considered "localizing" if it was lateralizing and all readers agreed on the same lobe within the same hemisphere. The impact of injection latency and post-injection seizure duration on the proportion of lateralizing/localizing SPECT was tested by ANOVA with dichotomized (by the median) injection latency and post-injection seizure duration as between-subjects factors.

**Results:**

Median [interquartile range] (full range) of injection latency and post-injection seizure duration were 30 [24, 40] (3–120) s and 50 [27, 70] (-20–660) s, respectively. Fleiss’ κ for lateralization of the SOZ was largest for the combination of early (< 30 s) injection and long (> 50 s) post-injection seizure duration (κ = 0.894, all other combinations κ = 0.659–0.734). Regarding Fleiss’ κ for localization of the SOZ in the 141 (80.1%) lateralizing SPECT, it was largest for early injection and short post-injection seizure duration (κ = 0.575, all other combinations κ = 0.329–0.368). The proportion of lateralizing SPECT was lower with short compared to long post-injection seizure duration (estimated marginal means 74.3% versus 86.3%, *p* = 0.047). The effect was mainly driven by cases with very short post-injection seizure duration ≤ 10 s (53.8% lateralizing). Injection latency in the considered range had no significant impact on the proportion of lateralizing SPECT (*p* = 0.390). The proportion of localizing SPECT among the lateralizing cases did not depend on injection latency or post-injection seizure duration (*p* ≥ 0.603).

**Conclusions:**

Short post-injection seizure duration is associated with a lower proportion of lateralizing cases in ictal brain perfusion SPECT.

**Supplementary Information:**

The online version contains supplementary material available at 10.1186/s13550-024-01095-5.

## Introduction

Epilepsy, one of the most prevalent neurological disorders, does not sufficiently respond to pharmacological treatment in a relevant proportion of the patients [1, 2]. Surgical resection of the epileptogenic zone offers a high chance of cure for individuals with focal epilepsy who do not become seizure-free under optimal drug therapy [1]. This requires reliable localization of the epileptogenic zone prior to surgery. If one of the conventional pointers to the epileptogenic zone assessed during presurgical evaluation (MRI, seizure semiology and electroencephalography (EEG)) is absent, or if there is discrepancy between these pointers, “more sophisticated imaging … is needed” [1].

The latter includes ictal brain perfusion single-photon emission computed tomography (SPECT) to identify the “seizure onset zone” (SOZ) [3, 4]. The ^99m^Tc-labeled tracers hexamethyl-propyleneamine oxime (^99m^Tc-HMPAO) [5, 6] and ethyl cysteinate dimer (^99m^Tc-ECD) [7–9] are widely used for this purpose. After intravenous bolus injection, the majority of the tracer is extracted from blood during a single capillary passage in the brain and locally retained in the adjacent brain tissue. Thus, regional tracer distribution within the brain mainly reflects the (relative) regional cerebral blood flow during the first capillary passage [10, 11]. The SPECT image can be considered a “snapshot” of the cerebral blood flow pattern at this time point [12].

When the tracer is injected during a seizure, the SOZ is identified by regional hyperperfusion [13]. This is complicated by the fact that “the scintigraphic appearance and extent of seizure foci may change dramatically depending on the exact timing of tracer injection relative to seizure onset” [14]. In particular, ipsilateral and contralateral seizure propagation [15, 16], surround inhibition [13] and postictal switch from hyper- to hypoperfusion in the SOZ [17] can have a major impact on the tracer uptake pattern depending on the delay of its intravenous injection relative to the start of the seizure [4]. The impact of the injection latency on the performance of ictal brain perfusion SPECT to correctly identify the epileptogenic zone has been investigated in numerous studies that consistently favored early injection [16, 18–26]. As a consequence, procedure guidelines for brain perfusion SPECT using ^99m^Tc-labelled radiopharmaceuticals recommend that “the tracer should be injected as soon as possible after seizure onset “ [27]. One of the main goals is to avoid relevant postictal switch effects, which typically occur not earlier than 60-90 s after the end of the seizure [21]. In contrast, seizure propagation effects cannot be ruled out even if the tracer injection starts simultaneously with the seizure. This is due to the approximately 15 s transit time of the tracer from the venous injection site to the brain [16, 28, 29]. Furthermore, the final tracer distribution in the brain is not fully complete until about 2 min after intravenous injection, mainly due to incomplete extraction of the tracer during a single capillary passage [30].

In striking contrast to the injection latency, the impact of the remaining seizure duration after the tracer injection on the performance of ictal perfusion SPECT is not well studied, although it appears plausible to assume that ictal neuronal activity and/or associated haemodynamic changes must still be ongoing when the tracer reaches the brain in order to identify the SOZ in the SPECT image. Procedure guidelines recommend that tracer injection should be exactly related “to the time point of behavioral and electrical seizure onset *and end”* [27], but they do not make any further statements concerning the injection time relative to the end of the seizure [14, 27].

Against this background, the aim of the current study was to assess the impact of the post-injection electrical seizure duration on the proportion of lateralizing and localizing ictal SPECT in a large clinical patient sample. The primary a priori hypothesis put to test was that short post-injection seizure duration is associated with a reduced proportion of ictal SPECT that allow reliable lateralization of the SOZ.

## Material and methods

### Patients

The database of the Department of Nuclear Medicine of the University Medical Center Hamburg-Eppendorf was searched for patients with suspected unifocal epilepsy who had undergone ictal brain perfusion SPECT with ^99m^Tc-HMPAO or ^99m^Tc-ECD for presurgical evaluation. The following inclusion criteria were employed: (I1) SPECT and structural MRI data available for consistent retrospective image processing, (I2) video EEG recording of the SPECT seizure available for retrospective analysis, (I3) clear identification of a seizure in the EEG that allowed reliable determination of (a) the latency of tracer injection after electrical seizure onset and (b) the remaining electrical duration of the seizure after tracer injection. These criteria were fulfilled by 184 (90.3%) of 207 screened patients. Of these, eight patients were excluded due to severe image artifacts caused by head motion during ictal SPECT acquisition. The remaining 176 patients were included into the analysis. No further eligibility criteria were employed. In particular, there were no eligibility criteria with respect to the suspected localization of the SOZ, with respect to MRI findings or with respect to prior therapy (including prior brain surgery). This was to guarantee that the included patient sample was representative of brain perfusion SPECT for presurgical evaluation of epilepsy patients in clinical routine at our site (tertiary referral epilepsy specialist center).

Most of the patients showed alterations in structural MRI that were taken into consideration as epileptogenic zone, including hippocampal sclerosis/atrophy (29.1%), border zone of prior surgery (13.6%), focal cortical dysplasia (10.9%), tumor (4.5%), post encephalitic lesion (1.8%), vessel malformation (1.8%) or unclear lesion (15.5%). The remaining 22.7% of patients presented with normal MRI. Demographic and clinical data of the included patients are summarized in Table 1.

**Tab 1:** Demographic and clinical data

	Median (IQR)	P-value of between-subjects effect		
		Injection latency	Seizure duration	Interaction
Age at ictal SPECT, y	38 (26–49)	0.052	0.403	0.997
Sex, % females	47.2	0.327	0.481	0.949
Age at first seizure, y	15 (7–24) (n = 64)	0.240	0.708	0.427
Duration of disease at SPECT, y	20 (10–31) (n = 65)	0.615	0.151	0.866
Mean seizure frequency in the last 12 mo before SPECT, seizures/mo	9 (5–22) (n = 50)	0.650	0.783	0.770
Tracer of ictal SPECT, % ^99m^Tc-ECD	20.5	0.978	0.051	0.445
Injected tracer dose, MBq	590 (505–700) (n = 115)	0.529	0.561	0.618
Delay between tracer injection and scan start, min	141 (100–188) (n = 93)	0.752	0.834	0.735

The ictal SPECT included in this study had been acquired between June 2004 and November 2021. The majority of the SPECT images had been included in a previous study on the impact of the tracer (^99m^Tc-HMPAO or ^99m^Tc-ECD) in ictal brain perfusion SPECT [31], a small subset had been included in a study on covariance pattern analysis of ictal brain perfusion SPECT for predicting the outcome of epilepsy surgery [32].

### Ictal SPECT

Ictal injection of ^99m^Tc-ECD (n = 36) or (stabilized) ^99m^Tc-HMPAO (n = 140) had been performed manually in an inpatient setting under scalp video-EEG monitoring in the Department of Neurology and Epileptology of the Protestant Hospital Alsterdorf. After tracer injection, the patients were transported to the Department of Nuclear Medicine of the University Medical Center Hamburg-Eppendorf for the SPECT acquisition (about 20 min drive).

SPECT projection data were acquired with a double-head camera (Siemens Symbia T2 or Siemens E.CAM) equipped with fan-beam or low-energy high-resolution collimators and angular steps of 2.8 or 3.0 degrees. The radius of rotation was 15.0 ± 1.3 cm. Total net acquisition time was 40 min.

The projection data were reconstructed into transaxial SPECT images with 3.9 × 3.9 × 3.9 mm^3^ voxel size using filtered backprojection implemented in the SPECT camera software with a Butterworth filter of order 5 and cutoff of 0.6 cycles/pixel (= 1.5 cycles/cm). The resulting images were postfiltered with an isotropic Gaussian kernel with 8 mm full-width-at-half-maximum. Uniform post-reconstruction attenuation correction was performed according to Chang (μ = 0.12/cm). No scatter correction was applied.

Consistent determination of the latency of the tracer injection after electrical seizure onset and of the remaining electrical seizure duration after tracer injection was performed retrospectively from the video EEG by experienced epileptologists. In the following, “seizure duration” always means *remaining* electrical seizure duration after tracer injection (= post-injection seizure duration).

### Image preprocessing

Each SPECT image was coregistered to the individual T1-weighted MRI. In addition, SPECT images were stereotactically normalized (affine) to the anatomical space of the Montreal Neurological Institute (MNI) using the statistical parametric mapping software package (version SPM12). Depending on the tracer, a custom ^99m^Tc-HMPAO template or a custom ^99m^Tc-ECD template in MNI space was used as target for the stereotactical normalization [31].

In preparation of voxel-based statistical testing for ictal hyperperfusion, stereotactically normalized SPECT images were smoothed by convolution with an isotropic 3-dimensional Gaussian kernel with 15 mm full-width-at-half-maximum and then scaled to the individual mean tracer uptake in a standard cerebrum parenchyma mask predefined in MNI space. The resulting images were transformed to z-score maps relative to the voxel-wise mean and the voxel-wise standard deviation of stereotactically normalized, smoothed and scaled tracer uptake in a custom ^99m^Tc-HMPAO normal database or a custom ^99m^Tc-ECD normal database, depending on the tracer used in the individual patient [32]. A rather conservative cutoff z ≥ 3.0 on the z-score was used to identify significant hyperperfusion. This is the standard cutoff used in clinical routine at our site in order to limit the rate of false positive clusters.

### Visual SPECT interpretation

**Fig. 1:**
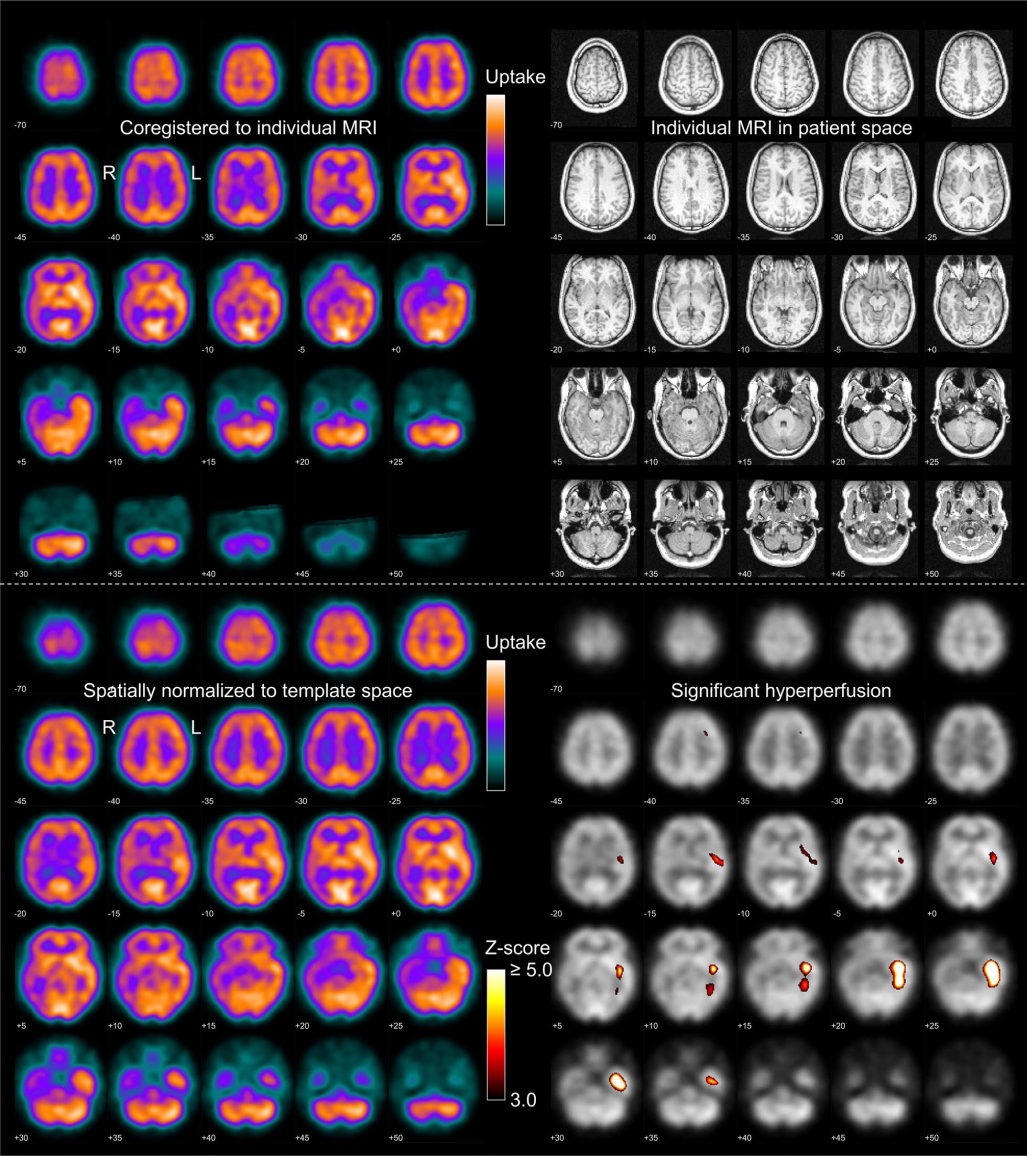
Standardized display for visual reading. The upper and lower parts were provided as page 1 and page 2 of a 2-page pdf-document (separate pdf-document for each patient). On the first page, the left side shows the patient’s ictal perfusion SPECT coregistered to the individual T1-weighted MRI shown on the right. On the second page, the left side shows the patient’s ictal perfusion SPECT after stereotactical normalization into the anatominal standard space, the righ side shows the statistical hyperperfusion map thresholded at a z-score of 3.0 and overlaid to the patient’s SPECT. This representative report is from a 16y old female with post-encephalitic lesion in the left temporal lobe. Ictal perfusion SPECT was performed with 500 MBq ^99m^Tc-ECD injected 19 s after electrical start of the seizure. The seizure continued for 41 s after the tracer injection. All three readers lateralized the SOZ in the left hemisphere and localized it to the left tempotal lobe, both with highest certainty. After surgical resection of the lesion, the subject was free of seizures (Engel IA) during the entire follow-up of 60 months

Visual interpretation of the ictal SPECT was based on a standardized display provided to the readers as a 2-page pdf-document (Fig. 1, a separate pdf-document was created for each patient).

The pseudonymized pdf documents were independently assessed by 3 readers (AK, KS, RB). The readers were blinded to all information except sex, age, and tracer.

The readers were asked to first lateralize the SOZ (right hemisphere, left hemisphere or no evidence of the SOZ). If the reader lateralized the SOZ to the right or to the left hemisphere, she/he was asked to characterize the certainty of the lateralization according to a 5-score (1 = very uncertain, …, 5 = very certain). Furthermore, the readers were asked to localize the SOZ within a brain lobe (temporal, frontal, parietal, occipital) in the selected hemisphere and to characterize the certainty of the localization according to the same 5-score (1 = very uncertain, …, 5 = very certain).

The score “no evidence of SOZ” at the first step was restricted to cases with normal tracer uptake. In cases with ≥ 2 SOZ candidates that were considered equally likely to be the actual SOZ, the readers were asked to select one SOZ candidate and to account for the uncertainty by a low certainty score for lateralization and/or localization.

The readers were asked to adhere to the criteria for visual interpretation of ictal SPECT given in the Additional file 1 Prior to the visual reading session, each reader performed a training session including 12 randomly selected cases.

### Statistical analysis

#### Definitions

An ictal SPECT was considered "lateralizing" if the 3 readers agreed on right or left hemisphere for the SOZ.

An ictal SPECT was considered "empty" (with respect to SOZ candidates) if at least 2 of the 3 readers categorized it as “no evidence of the SOZ”, that is, normal tracer uptake throughout the brain.

An ictal SPECT was considered "localizing" if it was “lateralizing” and the 3 readers also agreed on the same lobe within the same hemisphere.

Among the patients with “lateralizing” ictal SPECT who underwent epilepsy surgery in the hemisphere suggested by SPECT and in whom ≥ 12 months follow-up was available, those with Engel IA at 12 months were considered “responders”, all others were considered “non-responders”.

The tracer injection latency after the electrical start of the seizure was dichotomized using its median value: “early” injection if latency < median, “late” injection if latency ≥ median.

The post-injection electrical seizure duration, too, was dichotomized using its median value: “long” duration if > median, “short” duration if ≤ median.

#### Statistical tests

Proportions are given as percentage, continuous variables are given as median and interquartile range.

Spearman's rank correlation was employed to test for a potential association between injection latency and seizure duration (continuous variables).

Demographics (age, sex) and clinical variables (age at first seizure, duration of disease at ictal SPECT, mean seizure frequency in the last 12 months before SPECT, tracer, injected tracer dose, delay between tracer injection and start of the SPECT acquisition) were compared between the 4 subgroups regarding injection latency and post-injection seizure duration by using analysis of variance (ANOVA) with the demographic or clinical variable as dependent variable and dichotomized injection latency and dichotomized seizure duration as fixed between-subjects factors.

The impact of injection latency and seizure duration on the between-readers agreement of the visual SPECT interpretation was assessed by Fleiss’ kappa (κ). More precisely, Fleiss’ κ was used to characterize between-readers agreement of (i) the lateralization of the ictal SPECT (right hemisphere, left hemisphere, no evidence of the SOZ), and (ii) the localization of the SOZ (temporal, frontal, parietal, occipital) among the „lateralizing “ cases. This was performed in the whole sample as well as separately for each of the 4 combinations of „early “ versus „late “ injection and „long “ versus „short “ post-injection seizure duration. Statistical significance of pair-wise κ differences between subgroups was assessed by checking the 83.4% confidence intervals (CI) of the corresponding κ estimates for overlap (non-overlapping 83.4%-CI indicating statistical significance with 5% type 1 error probability) [33]. The 83.4%-CI was computed as: κ ± 1.385 * standard error of the κ estimate [34]. The interpretation of Fleiss ‘ κ values with respect to the strength of between-readers agreement was according to Landis and Koch [35].

The impact of injection latency and seizure duration on the proportion of “lateralizing” (“localizing”) ictal SPECT was tested by ANOVA with “lateralizing” (”localizing”) (yes/no) as dependent variable and dichotomized injection latency and dichotomized seizure duration as fixed between-subjects factors.

For more detailed testing of the impact of the seizure duration on the proportion of “lateralizing” ictal SPECT, the seizure duration was subdivided into 6 categories: <  = 10 s, 11-30 s, 31-50 s, 51-70 s, 71-90 s, amd > 90 s. Pearson’s χ2 test was used to compare the proportion of “lateralizing” ictal SPECT between these categories.

The impact of injection latency and seizure duration on the certainty of the lateralization (localization) amongst the “lateralizing” (“localizing”) ictal SPECT was tested by ANOVA with lateralization (localization) certainty (mean across all readers) as dependent variable and dichotomized injection latency and seizure duration as fixed between-subjects factors.

The impact of injection latency and seizure duration on surgery outcome was tested by ANOVA with outcome (responder/non-responder) as dependent variable and dichotomized injection latency and dichotomized seizure duration as fixed between-subjects factors.

Post-injection seizure duration was the between-subjects factor of primary interest in all statistical tests. The dichotomized injection latency was included as between-subjects factor in all models mainly to account for putative interaction effects.

The statistical analysis was performed with SPSS (version 29). An effect was considered statistically significant if two-sided *p* < 0.05.

## Results

Median [interquartile range] (full range) of injection latency and post-injection seizure duration were 30 [24, 40] (3–120) s and 50 [27, 70] (− 20–660) s, respectively. Injection latency and seizure duration were not correlated (Spearman’s rho = 0.025, *p* = 0.740). Thus, dichotomization of injection latency and seizure duration resulted in rather balanced subgroups (early injection/long duration, early/short, late/long, late/short: n = 41, 41, 45, 49).

The 4 subgroups with respect to injection latency and seizure duration did not differ significantly regarding any of the demographic or clinical variables (all *p* ≥ 0.051, Table 1).

### Lateralization of the SOZ

In the whole sample (n = 176), Fleiss’ κ with respect to lateralization of the SOZ (left hemisphere, right hemisphere, no evidence of the SOZ) was 0.736 (standard error 0.039, 83.4%-CI 0.688–0.790), indicating substantial agreement. Regarding the individual categories for lateralization, κ was significantly higher for lateralizing into a hemisphere than for categorizing the SPECT as “no evidence of the SOZ” (right hemisphere: κ = 0.769, 83.4%-CI 0.715–0.830, left hemisphere: κ = 0.791, 83.4%-CI 0.737–0.852, no evidence: κ = 0.126, 83.4%-CI 0.072–0.187).

**Fig. 2:**
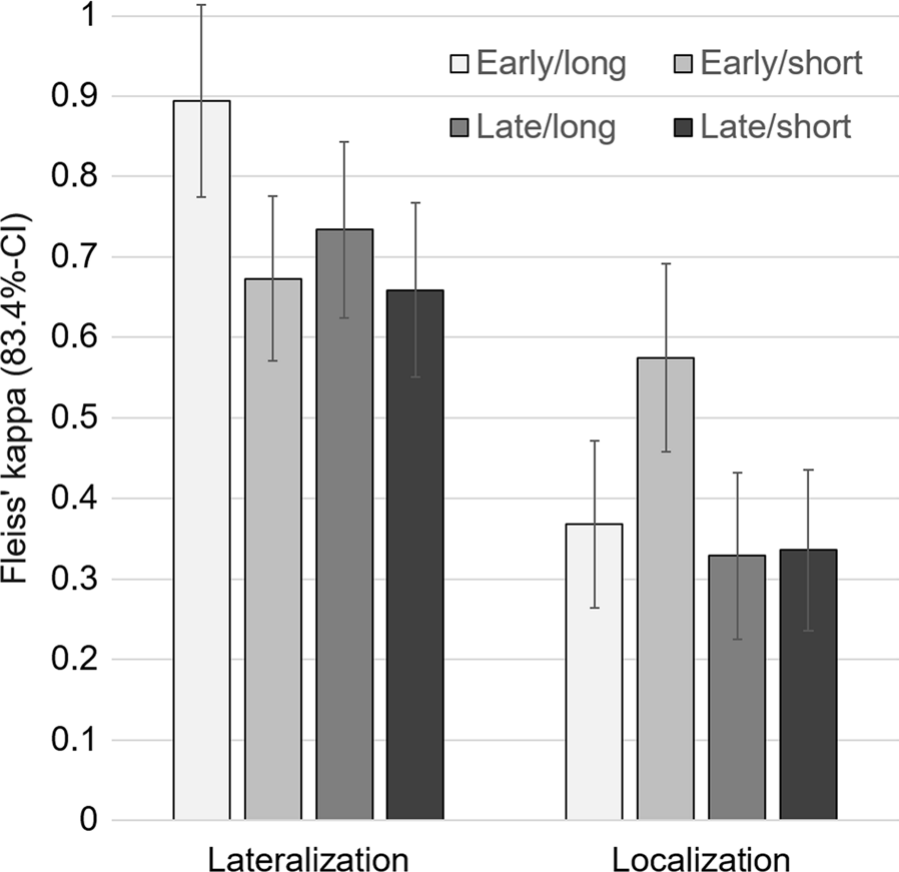
Between-readers agreement. Fleiss’ κ with respect to the lateralization of the SOZ, and with respect to the localization of the SOZ in a given lobe in the “lateralizing” cases. The error bars represent the 83.4%-CI. Non-overlapping 83.4%-CI indicate significantly different κ

Fleiss’ κ estimates of lateralization according to subgroups with respect to injection latency and seizure duration are summarized in Fig. 2. Between-readers agreement of lateralization was best for the combination of “early injection” and “long seizure duration”: κ = 0.894 (83.4%-CI 0.775–1.0), indicating almost perfect agreement. The difference was statistically significant compared to both subgroups with short seizure duration (non-overlapping 83.4%-CI, Fig. 2). The difference compared to late injection and long seizure duration was not significant.

The ictal SPECT was “lateralizing” in 141 of the 176 cases (80.1%).

**Fig. 3:**
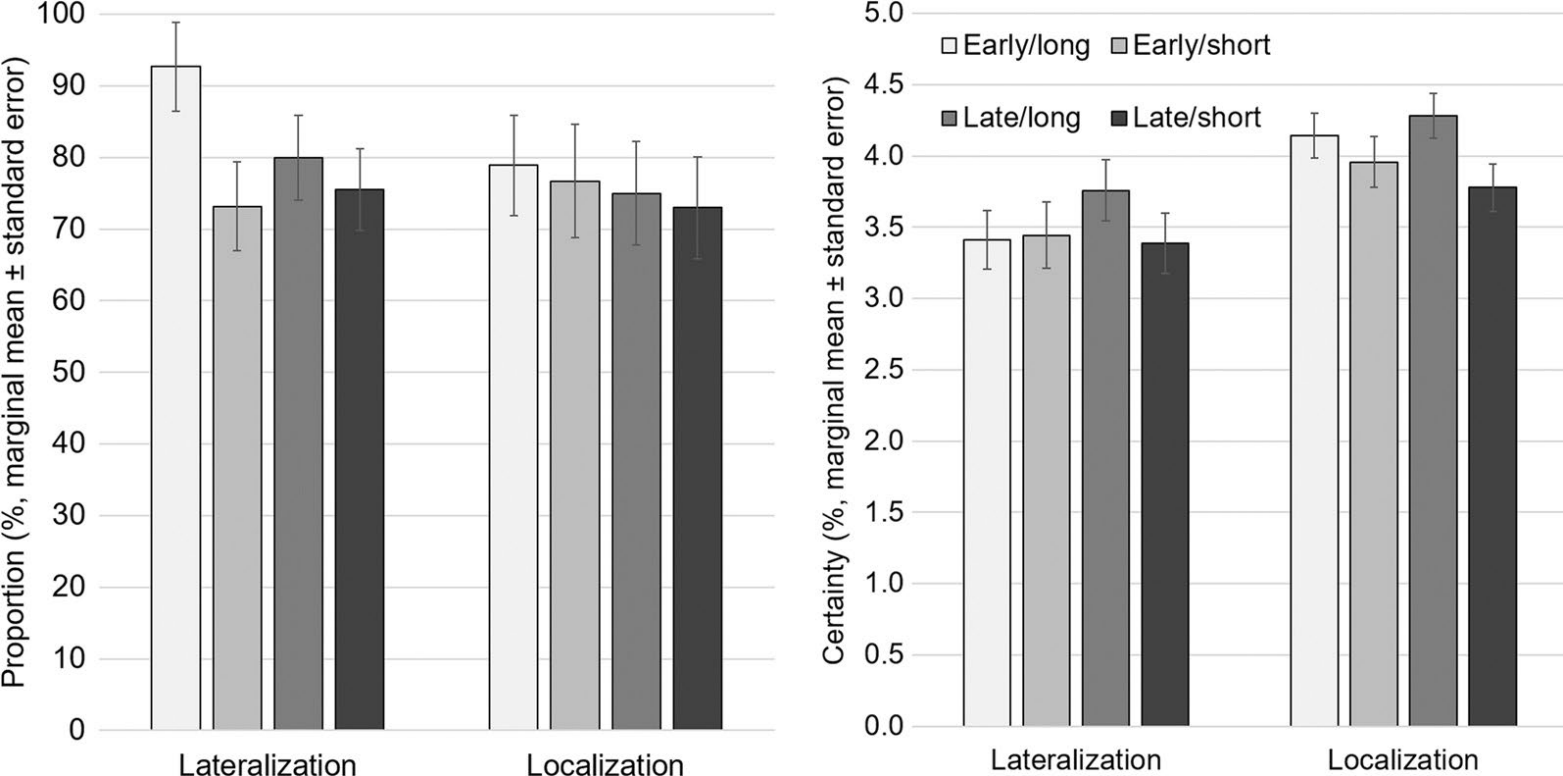
Lateralization and localization of the seizure onset zone. Proportion of “lateralizing” cases among all 176 ictal SPECT and proportion of “localizing” cases among the 141 lateralizing ictal SPECT (left), and certainty of the lateralization among the 141 “lateralizing” cases and certainty of the localization among the 107 “localizing” cases (right). The bars represent marginal means estimated by ANOVA with dichotomized injection latency (“early” versus “late” injection) and dichotomized post-injection seizure duration (“long” versus “short” duration) as fixed between-subjects factors. The error bars represent the standard error of the marginal means. The results are shown separately for each combination of dichotomized injection latency and dichotomized post-injection seizure duration

The proportion of “lateralizing” SPECT did not depend on the injection latency in the considered range (*p* = 0.390). In contrast, seizure duration had a significant effect: the proportion of “lateralizing” SPECT was higher with long compared to short seizure duration: estimated marginal means 86.3% versus 74.3% (*p* = 0.047). The increase of the proportion of “lateralizing” cases associated with “long” seizure duration compared to “short” seizure duration was more pronounced with “early” injection (from 73.2% to 92.7%, estimated marginal means) than with “late” injection (from 75.5% to 80.0%, Fig. 3). However, the latency*duration interaction effect did not reach statistical significance (*p* = 0.212).

The certainty of the lateralization among the “lateralizing” cases did not differ between subgroups (between-subjects effect of injection latency/seizure duration: *p* = 0.504/*p* = 0.434, Fig. 3).

**Fig. 4:**
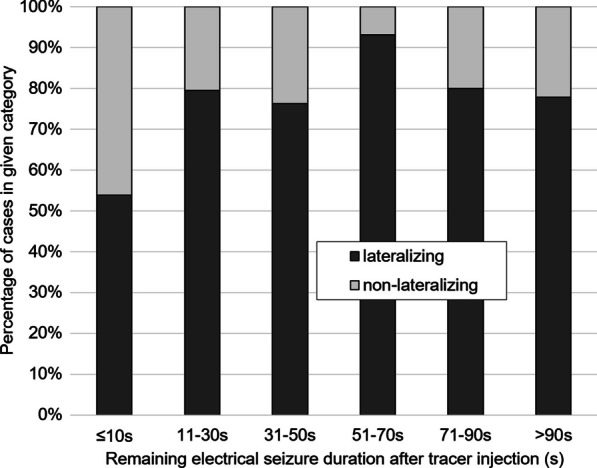
Lateralization of the seizure onset zone. Stacked histogram of the proportion of “lateralizing” versus “non-lateralizing” ictal SPECT for varying seizure duration

The proportion of “lateralizing” ictal SPECT according to seizure duration subdivided into 6 categories is shown in Fig. 4 (*p* = 0.056). The proportion of “lateralizing” SPECT was the lowest for seizure duration ≤ 10 s (53.8%).

The ictal SPECT was “empty” (normal cerebral blood flow) in 3 cases (1.7%). All “empty” cases were characterized by “early” injection and short post-injection seizure duration (5-10 s).

### Localization of the SOZ

Among the 141 lateralizing cases, Fleiss’ κ with respect to localization of the SOZ in a given lobe was 0.417 (standard error 0.037, 83.4%-CI 0.366–0.468), indicating fair to moderate agreement. Fleiss’ κ was largest for localization in the temporal lobe (0.497, 83.4%-CI 0.429–0.565), smallest for localization in the frontal lobe (0.281, 83.4%-CI 0.213–0.349), it was in between for localization in the parietal lobe (0.379, 83.4%-CI 0.311–0.447) or in the occipital lobe (0.329, 83.4%-CI 0.261–0.397).

Between-readers agreement of localization was best for the combination of “early” injection and “short” seizure duration: κ = 0.575 (83.4%-CI 0.459–0.691), indicating moderate to substantial agreement. The difference was statistically significant compared to both subgroups with “late” tracer injection (non-overlapping 83.4%-CI, Fig. 2). The difference compared to “early injection” and “long seizure duration” reached borderline significance (minor overlap of the 83.4%-CI).

The ictal SPECT was “localizing” in 107 of the 141 lateralizing cases (75.9%, 60.8% of all 176 cases). The vast majority of the “localizing” ictal SPECT was localizing to the temporal lobe (96.3%). The remaining “localizing” SPECT were localizing to the parietal lobe (2.8%) or to the frontal lobe (0.9%).

The proportion of “localizing” SPECT was highest amongst the “lateralizing” SPECT with “early” tracer injection and “long” seizure duration (Fig. 3). However, neither the effect of injection latency (*p* = 0.603) nor the effect of seizure duration (*p* = 0.769) on the proportion of “localizing” cases was statistically significant.

The certainty of the localization among the “localizing” cases did not depend on injection latency (*p* = 0.907). In contrast, seizure duration had a significant effect: the certainty of the localization was higher with long seizure duration: 4.21 versus 3.87 (estimated marginal means, *p* = 0.040, Fig. 3).

### Surgery outcome

Among the patients with “lateralizing” ictal SPECT who underwent epilepsy surgery in the hemisphere suggested by SPECT, and for whom ≥ 12 months follow-up was available (n = 27), 11 (40.7%) were classified as “responder”, while the remaining 16 (59.3%) were classified as “non-responder”.

There was a trend towards a significant effect of injection latency: the proportion of responders was higher with “early” compared to “late” injection: 66.7% versus 28.7% (estimated marginal means, *p* = 0.081). Seizure duration did not have a significant effect (*p* = 0.677).

## Discussion

The main finding of the current study was a significantly reduced proportion of ictal brain perfusion SPECT that allowed consistent (across all readers) lateralization of the SOZ when the post-injection seizure duration was short, in line with the a priori hypothesis of the study. This effect was mainly driven by cases with very short (≤ 10 s) post-injection seizure duration. This was corroborated by the fact that all “empty” ictal SPECT (normal tracer uptake in the brain according to at least 2 of the 3 readers) were characterized by very short post-injection seizure duration (5–10 s). Concerning the localization of the SOZ in a given brain lobe, the proportion of cases with consistent (across all readers) localization amongst the lateralizing cases was the highest for the combination of early injection and long post-injection seizure duration, again favoring long post-injection seizure duration, although the effect did not reach statistical significance.

These findings are in agreement with previous studies that mostly showed that the optimal post-injection seizure duration for ictal brain perfusion SPECT being able to identify the SOZ is > 20–30 s [19, 29]. Aungaroon and co-workers performed subtraction ictal and interictal SPECT with ^99m^Tc-ECD or ^99m^Tc-HMPAO coregistered to MRI (SISCOM) [36] in 113 children with drug-resistant epilepsy [19]. Injection latency ranged from 2 to 260 s, post-injection seizure duration ranged from 0 to 631 s. A SISCOM was considered “localizing” if at least two of three readers agreed on the same hyperperfusion area. The odds of a SISCOM being localizing increased by 0.7% for every 1 s post-injection seizure duration (*p* = 0.06). Additional analyses using multiple cutoffs revealed that this improvement was mainly driven by a step at about 20 s post-injection seizure duration [19]. Prener and co-workers compared the performance of SISCOM with ^99m^Tc-HMPAO between 39 patients with post-injection seizure duration ≥ 30 s (mean 76 s, range 30–276 s) and 17 patients with post-injection seizure duration < 30 s (mean 13 s, range 0–28 s) [29]. Using the surgical resection area as gold standard, SISCOM was correctly localizing in 44% of the patients with ≥ 30 s post-injection seizure duration versus 35% in the patients with < 30 s post-injection seizure duration. The authors concluded “that seizure durations of less than 30 s after tracer injection lead to less consistent interpretations” [29, 37].

In view of approximately 15 s transit time of the tracer to reach the brain after intravenous injection [16, 28, 29], the current study together with these previous studies suggests that for best performance of ictal brain perfusion SPECT, post-injection seizure duration should be long enough to ensure that the seizure-related neuronal activity is still ongoing when the tracer reaches the brain.

Changes in electrical neuronal activity (as detected by EEG) and haemodynamic changes (as measured by brain perfusion SPECT) are tightly linked by neurovascular coupling: increased neuronal activity is associated with increased local energy demand and therefore triggers an increase of local blood flow to provide additional oxygen and glucose [4, 38–41]. Neurovascular coupling is active not only during normal somatosensory processing, but also in pathological states including epilepsy, although it is probably not sufficient to fully match the far supranormal local energy demands during a seizure [42, 43]. However, neurovascular coupling does not imply that the temporal scales of changes in electrical neuronal activity and the resulting changes in cerebral perfusion are the same. In particular, is does not rule out longer lasting perfusion changes after the electrical EEG activity associated with an epileptic seizure has returned to normal.

Data on the duration of hyperperfusion at the seizure focus is limited. Based on the fact that the brain is generally warmer than incoming blood, so that increases in blood flow cause cooling in the brain, Dymond and Crandall used intracerebral electrodes to measure temperature changes at the seizure focus as an index of blood flow alteration during spontaneous complex partial seizures in 7 patients [44]. They concluded from their findings that the regional cerebral blood flow in the seizure focus “seems to be stabilized again after 10–15 min” [44]. Brodersen and co-workers, using the ^133^Xe clearance method to measure cerebral blood flow in an anesthetized, paralyzed, and ventilated man during electrically induced seizures, found the seizure-associated flow changes to normalize within 2 min after the end of the seizure [45]. Zhao and co-workers, recording intrinsic optical signals from human cortex intraoperatively during 3 spontaneous seizures arising from brain surrounding a small cavernous malformation in an awake patient, found the haemodynamic changes to persist for at least 1–2 min after the offset of the seizure [38, 42]. These findings suggest that ictal hyperperfusion in the SOZ might last between one and several minutes after normalization of the EEG signal, which might allow identification of the SOZ by regional hyperperfusion in ictal brain perfusion SPECT also with postictal tracer injection. This was confirmed by Rowe and co-workers who investigated the power of ^99m^Tc-HMPAO SPECT with postictal tracer injection for the localization of epileptic foci [18]. Postical injection was on average 5 min after seizure start. All seizures lasted less than 2 min, and no patient had ongoing ictal EEG activity at the time of injection. Roughly half of the patients (19/36), in which HMPAO was injected more than 3 min after the start of the seizure, had an appropriate focal change in the postictal SPECT, indicating prolonged hyperperfusion in the SOZ. However, almost three-quarters of patients (8/11) showed an appropriate focal change in the postictal SPECT when HMPAO was injected within 3 min of the start of the seizure [18]. In 2 patients, repeat studies with shorter postictal injection delay showed focal hyperperfusion not seen on the first scan [18]. In 3 patients who had multiple post-ictal studies it was noted that mesial temporal hyperperfusion decreased with increasing time from seizure onset to injection [18]. In another study that compared “ictal” injection (during the seizure or up to 30 s after its electroclinical termination) and postictal injection (30 s or more after seizure termination) in ^99m^Tc-HMPAO SPECT in patients with temporal lobe epilepsy, the epileptogenic focus was correctly lateralized in 97% of the ictal studies versus 72% in the postictal studies (5% of the postictal studies lateralized to the wrong hemisphere) [21]. These findings suggest that brain perfusion SPECT with postictal tracer injection can be useful but is associated not only with reduced sensitivity for detection of the SOZ but also with increased risk of false lateralization. It might be noted in this context that “normalization” of ictal hyperperfusion in the SOZ in most studies means return to the interictal level. If the interictal blood flow is reduced in the SOZ [46], the ictal perfusion might fall below the threshold for the detection of hyperperfusion in the ictal SPECT image alone, either by visual inspection or by voxel-based statistical testing against a normal database, before ictal hyperperfusion returns to the interictal level.

Zubal and co-workers assessed ictal perfusion changes in the SOZ as calculated from the difference of ictal and interictal ^99m^Tc-HMPAO SPECT in relation to seizure duration and the time of tracer injection [16]. The study included 19 patients with seizure duration ranging from 2 to 275 s and injection time ranging from 96 s before to 100 s after termination of the seizure [16]. The authors proposed 3 general categories to explain the cross-sectional perfusion changes observed in their study: (i) ictal hyperperfusion associated with ictal injections accomplished well prior to seizure termination, (ii) excessive hypoperfusion, lasting for a period of about one-third of the seizure duration, for ictal injections immediately after seizure termination, and (iii) persistent hypoperfusion when the ictal injection was completed several tens of seconds after seizure cessation [16]. They concluded that the time of injection relative to the time of seizure cessation is more relevant regarding the interpretation of ictal brain perfusion SPECT than the latency of tracer injection relative to the seizure onset [16]. In particular, short lasting but excessive hypoperfusion in the SOZ shortly after seizure termination might contribute to reduced sensitivity of ictal perfusion SPECT with too short post-injection seizure duration. The short lasting but excessive hypoperfusion in the SOZ shortly after seizure termination described by Zubal and co-workers might differ from the postictal switch phenomenon that typically occurs not earlier than 60-90 s after the end of the seizure [17, 21].

In a study on the spatiotemporal dynamics of perfusion associated with epileptic seizures in the rat neocortex, the duration of the blood flow increase in the seizure focus was directly correlated with the seizure duration [47]. This implies that the recovery from a long seizure takes more time than that from a short seizure [16].

Dupont and co-workers investigated the temporal relationship of ictal perfusion changes with injection time and seizure duration in brain perfusion SPECT with ^99m^Tc-ECD in 37 patients with temporal lobe epilepsy [15]. The injection latency after seizure onset ranged between 13 and 60 s, remaining seizure duration following tracer injection ranged between 20 and 881 s. There was a positive correlation of the percent perfusion change in the ictal SPECT compared to the interictal SPECT with the remaining seizure duration in the ipsilateral putamen extending to the ipsilateral orbitofrontal cortex. The correlation was driven by more or less normal ictal perfusion with short seizure duration that switched to marked hyperperfusion at long seizure duration. A negative correlation of the ictal perfusion change with the remaining seizure duration was observed in the bilateral superior frontal lobe and in the precuneus. In both regions, short seizure duration was associated with mild ictal hyperperfusion, long seizure duration was associated with mild ictal hypoperfusion. The pronounced ictal hyperperfusion observed in the ipsilateral temporal lobe, the SOZ, was not significantly correlated with the seizure duration. Nevertheless, the findings of this study should be taken into account when interpreting the complex ictal perfusion patterns regarding lateralization and localization of the SOZ.

Strengths of the current study include the rather large number of ictal brain perfusion SPECT with clear seizure identification in video EEG and the systematic retrospective analysis, both of the video EEG for reliable determination of injection latency and electrical post-injection seizure duration, and of the ictal SPECT images. Furthermore, 12 months follow-up after epilepsy surgery was available in a sufficiently large number of patients to allow testing the impact of the post-injection seizure duration on the power of ictal SPECT to predict the surgical outcome.

The following limitations of the study should be noted. First, the vast majority of the “localizing” ictal SPECT localized the SOZ to the temporal lobe suggesting that the majority of the included patients had temporal lobe epilepsy. Thus, the findings might not be translated without change to extratemporal epilepsies, given that extratemporal seizures are shorter [48] and postictal switch might occur faster after seizure termination [17] than in temporal epilepsies. Second, age was not taken into account as nuisance covariate (of no interest) in the voxel-based statistical testing for ictal hyperperfusion. This might have affected the statistical hyperperfusion maps provided to the readers to support visual interpretation of the ictal SPECT, since the normal pattern of cerebral blood flow as measured by brain perfusion SPECT depends on age, even after global intensity scaling of the regional tracer uptake (e.g., relatively more pronounced age-related decline in the medial frontal cortex) [49]. However, this most likely did not affect the main conclusions from this study, because the 4 subgroups with respect to injection latency and seizure duration did not differ significantly regarding any of the demographic or clinical variables including age. Finally, the visual analysis of the ictal SPECT images adhered to the common procedure guidelines for brain perfusion SPECT in epilepsy [14, 27, 50–52] except for the use of a standardized pdf-document rather than a computer monitor. Thus interactive manipulations such as changing the color table or the image orientation was not possible. The rationale for the use of the pdf-documents was to standardize the reading across the readers. Furthermore, different than in practice, the readers were intentionally blinded to clinical and EEG data in order to avoid potential bias.

## Conclusions

The results of the current study indicate a significant impact of the post-injection seizure duration on the performance of ictal brain perfusion SPECT. Specifically, short electrical post-injection seizure duration is associated with a higher likelihood of non-lateralizing ictal SPECT. This finding might have a relevant impact on clinical practice. First, tracer injection might be avoided if the seizure has already terminated. Second, the lack of a candidate for the SOZ on ictal perfusion SPECT is less conclusive if the post-injection seizure duration was short, which might justify to repeat the ictal SPECT [53]. Furthermore, it is evident that long injection latency is associated with an increased risk of too short post-injection seizure duration. Thus, the current findings regarding the impact of the post-injection seizure duration provide independent support of the recommendation to inject the tracer as soon as possible after seizure onset [27]. This might be achieved by specific training of the manual injection [54] or by the use of an automatic injection system [20, 55].

### Supplementary Information

**Additional file 1**. Criteria for the visual interpretation of ictal SPECT.

## Data Availability

The datasets (visual scores) used and/or analysed during the current study as well as the custom MATLAB/SPM12 script for fully automatic processing of ictal SPECT images including voxel-based statistical testing are available from the last author upon reasonable request.

## Abbreviations

^99m^Tc-ECD: 99M Tc-labeled ethyl cysteinate dimer
^99m^Tc-HMPAO: 99M Tc-labeled hexamethyl-propyleneamine oxime
ANOVA: Analysis of Variance
CI: Confidence interval
EEG: Electroencephalography
MNI: Montreal Neurological Institute
MRI: Magnetic resonance imaging
SOZ: Seizure onset zone
SPECT: Single-photon emission computed tomography
SPM12: Statistical Parametric Mapping software package, version 12
κ: Fleiss’ kappa

## References

[1] West S, Nolan SJ, Cotton J, Gandhi S, Weston J, Sudan A (2015). Surgery for epilepsy. Cochrane Database Syst Rev.

[2] Kwan P, Brodie MJ (2000). Early identification of refractory epilepsy. N Engl J Med.

[3] Blend MJ, de Leon OA, Jobe TH, Lin Q, Sychra JJ, Gaviria M (1997). Cerebral perfusion SPECT imaging in epileptic and nonepileptic seizures. Clin Nucl Med.

[4] Van Paesschen W (2004). Ictal SPECT. Epilepsia.

[5] Sharp PF, Smith FW, Gemmell HG, Lyall D, Evans NT, Gvozdanovic D (1986). Technetium-99m HM-PAO stereoisomers as potential agents for imaging regional cerebral blood flow: human volunteer studies. J Nucl Med.

[6] Neirinckx RD, Canning LR, Piper IM, Nowotnik DP, Pickett RD, Holmes RA (1987). Technetium-99m d, l-HM-PAO: a new radiopharmaceutical for SPECT imaging of regional cerebral blood perfusion. J Nucl Med.

[7] Leveille J, Demonceau G, De Roo M, Rigo P, Taillefer R, Morgan RA (1989). Characterization of technetium-99m-L, L-ECD for brain perfusion imaging, Part 2: biodistribution and brain imaging in humans. J Nucl Med.

[8] Walovitch RC, Hill TC, Garrity ST, Cheesman EH, Burgess BA, O'Leary DH (1989). Characterization of technetium-99m-L, L-ECD for brain perfusion imaging, Part 1: pharmacology of technetium-99m ECD in nonhuman primates. J Nucl Med.

[9] Vallabhajosula S, Zimmerman RE, Picard M, Stritzke P, Mena I, Hellman RS (1989). Technetium-99m ECD: a new brain imaging agent: in vivo kinetics and biodistribution studies in normal human subjects. J Nucl Med.

[10] Colamussi P, Calo G, Sbrenna S, Uccelli L, Bianchi C, Cittanti C (1999). New insights on flow-independent mechanisms of 99mTc-HMPAO retention in nervous tissue: in vitro study. J Nucl Med.

[11] Neirinckx RD, Burke JF, Harrison RC, Forster AM, Andersen AR, Lassen NA (1988). The retention mechanism of technetium-99m-HM-PAO: intracellular reaction with glutathione. J Cereb Blood Flow Metab.

[12] O'Brien TJ, Zupanc ML, Mullan BP, O'Connor MK, Brinkmann BH, Cicora KM (1998). The practical utility of performing peri-ictal SPECT in the evaluation of children with partial epilepsy. Pediatr Neurol.

[13] Schwartz TH, Bonhoeffer T (2001). In vivo optical mapping of epileptic foci and surround inhibition in ferret cerebral cortex. Nat Med.

[14] Juni JE, Waxman AD, Devous MD, Tikofsky RS, Ichise M, Van Heertum RL (2009). Procedure guideline for brain perfusion SPECT using Tc-99m radiopharmaceuticals 3.0. J Nucl Med Technol.

[15] Dupont P, Zaknun JJ, Maes A, Tepmongkol S, Vasquez S, Bal CS (2009). Dynamic perfusion patterns in temporal lobe epilepsy. Eur J Nucl Med Mol Imaging.

[16] Zubal IG, Spanaki MV, MacMullan J, Corsi M, Seibyl JP, Spencer SS (1999). Influence of technetium-99m-hexamethylpropylene amine oxime injection time on single-photon emission tomography perfusion changes in epilepsy. Eur J Nucl Med.

[17] Newton MR, Berkovic SF, Austin MC, Rowe CC, Mckay WJ, Bladin PF (1992). Postictal Switch in blood-flow distribution and temporal-lobe seizures. J Neurol Neurosur Ps.

[18] Rowe CC, Berkovic SF, Sia STB, Austin M, Mckay WJ, Kalnins RM (1989). Localization of epileptic foci with postictal single photon-emission computed-tomography. Ann Neurol.

[19] Aungaroon G, Trout A, Radhakrishnan R, Horn PS, Arya R, Tenney JR (2018). Impact of radiotracer injection latency and seizure duration on subtraction ictal SPECT co-registered to MRI (SISCOM) performance in children. Clin Neurophysiol.

[20] Setoain X, Campos F, Donaire A, Mayoral M, Perissinotti A, Ninerola-Baizan A (2021). How to inject ictal SPECT? From manual to automated injection. Epilepsy Res.

[21] Newton MR, Berkovic SF, Austin MC, Rowe CC, Mckay WJ, Bladin PF (1994). Ictal postictal and interictal single-photon emission tomography in the lateralization of temporal-lobe epilepsy. Eur J Nucl Med.

[22] Lee SK, Lee SY, Yun CH, Lee HY, Lee JS, Lee DS (2006). Ictal SPECT in neocortical epilepsies: clinical usefulness and factors affecting the pattern of hyperperfusion. Neuroradiology.

[23] Lee JY, Joo EY, Park HS, Song P, Byun SY, Seo DW (2011). Repeated ictal SPECT in partial epilepsy patients: SISCOM analysis. Epilepsia.

[24] Kahane P, Merlet I, Grégoire MC, Munari C, Perret J, Mauguière F (1999). An H215 O-PET study of cerebral blood flow changes during focal epileptic discharges induced by intracerebral electrical stimulation. Brain.

[25] Herholz K, Teipel S, Hellwig S, Langner S, Rijntjes M, Klöppel S, et al. Functional and Molecular Neuroimaging. In: Jankovic J, Mazziotta JC, Pomeroy SL, Newman NJ, editors. Neurology in Clinical Practice. 8th ed: Elsevier; 2022.

[26] Avery RA, Spencer SS, Spanaki MV, Corsi M, Seibyl JP, Zubal IG (1999). Effect of injection time on postictal SPET perfusion changes in medically refractory epilepsy. Eur J Nucl Med.

[27] Kapucu OL, Nobili F, Varrone A, Booij J, Vander Borght T, Nagren K (2009). EANM procedure guideline for brain perfusion SPECT using Tc-99m-labelled radiopharmaceuticals, version 2. Eur J Nucl Med Mol.

[28] Knudsen GM, Pettigrew KD, Patlak CS, Paulson OB (1994). Blood-brain barrier permeability measurements by double-indicator method using intravenous injection. Am J Physiol.

[29] Prener M, Drejer V, Ziebell M, Jensen P, Madsen CG, Olsen S (2023). Ictal and interictal SPECT with Tc-99m-HMPAO in presurgical epilepsy. I: Predictive value and methodological considerations. Epilepsia Open..

[30] Andersen AR, Friberg H, Knudsen KB, Barry DI, Paulson OB, Schmidt JF (1988). Extraction of [99mTc]-d, l-HM-PAO across the blood-brain barrier. J Cereb Blood Flow Metab.

[31] Jaber M, Taherpour J, Voges B, Apostolova I, Sauvigny T, House PM (2021). No evidence to favor 99mTc-HMPAO or 99mTc-ECD for Ictal brain perfusion SPECT for identification of the seizure onset zone. Clin Nucl Med.

[32] Taherpour J, Jaber M, Voges B, Apostolova I, Sauvigny T, House PM (2022). Predicting the outcome of epilepsy surgery by covariance pattern analysis of ictal perfusion SPECT. J Nucl Med.

[33] Knol MJ, Pestman WR, Grobbee DE (2011). The (mis)use of overlap of confidence intervals to assess effect modification. Eur J Epidemiol.

[34] Zapf A, Castell S, Morawietz L, Karch A (2016). Measuring inter-rater reliability for nominal data - which coefficients and confidence intervals are appropriate?. BMC Med Res Methodol.

[35] Landis JR, Koch GG (1977). The measurement of observer agreement for categorical data. Biometrics.

[36] O'Brien TJ, So EL, Mullan BP, Hauser MF, Brinkmann BH, Bohnen NI (1998). Subtraction ictal SPECT co-registered to MRI improves clinical usefulness of SPECT in localizing the surgical seizure focus. Neurology.

[37] Prener M, Drejer V, Ziebell M, Jensen P, Madsen CG, Olsen S (2023). Ictal and interictal SPECT with (99m) Tc-HMPAO in presurgical epilepsy. II: Methodological considerations on hyper- and hypoperfusion. Epilepsia Open..

[38] Schwartz TH, Hong SB, Bagshaw AP, Chauvel P, Bénar CG (2011). Preictal changes in cerebral haemodynamics: Review of findings and insights from intracerebral EEG. Epilepsy Res.

[39] Lauritzen M, Gold L (2003). Brain function and neurophysiological correlates of signals used in functional neuroimaging. J Neurosci.

[40] Raichle ME, Mintun MA (2006). Brain work and brain imaging. Annu Rev Neurosci.

[41] Logothetis NK, Wandell BA (2004). Interpreting the BOLD signal. Annu Rev Physiol.

[42] Zhao MR, Suh MA, Ma HT, Perry C, Geneslaw A, Schwartz TH (2007). Focal increases in perfusion and decreases in hemoglobin oxygenation precede seizure onset in spontaneous human epilepsy. Epilepsia.

[43] Girouard H, Iadecola C (2006). Neurovascular coupling in the normal brain and in hypertension, stroke, and Alzheimer disease. J Appl Physiol.

[44] Dymond AM, Crandall PH (1976). Oxygen availability and blood-flow in temporal lobes during spontaneous epileptic seizures in man. Brain Res.

[45] Brodersen P, Paulson OB, Bolwig TG, Rogon ZE, Rafaelsen OJ, Lassen NA (1973). Cerebral hyperemia in electrically induced epileptic seizures. Arch Neurol.

[46] Devous MD, Thisted RA, Morgan GF, Leroy RF, Rowe CC (1998). SPECT brain imaging in epilepsy: a meta-analysis. J Nucl Med.

[47] Zhao MR, Ma HT, Suh M, Schwartz TH (2009). Spatiotemporal dynamics of perfusion and oximetry during ictal discharges in the rat neocortex. J Neurosci.

[48] Kaminska A, Chiron C, Ville D, Dellatolas G, Hollo A, Cieuta C (2003). Ictal SPECT in children with epilepsy: comparison with intracranial EEG and relation to postsurgical outcome. Brain.

[49] Van Laere K, Versijpt J, Audenaert K, Koole M, Goethals I, Achten E (2001). 99mTc-ECD brain perfusion SPET: variability, asymmetry and effects of age and gender in healthy adults. Eur J Nucl Med.

[50] Kaewchur T, Chamroonrat W, Thientunyakit T, Khiewvan B, Wongsurawat N, Chotipanich C, et al. Thai National Guideline for Nuclear Medicine Investigations in Epilepsy. Asia Ocean J Nucl Med Biol. 2021;9. 10.22038/AOJNMB.2021.54567.1379 PMC8255518 34250150

[51] Garibotto V, Picard F (2013). Nuclear medicine imaging in epilepsy. Epileptologie.

[52] Ponisio MR, Zempel JM, Day BK, Eisenman LN, Miller-Thomas MM, Smyth MD (2021). The role of SPECT and PET in epilepsy. Am J Roentgenol.

[53] Lee DS, Lee SK, Kim YK, Kang E, Lee JS, Chung JK (2002). The usefulness of repeated ictal SPET for the localization of epileptogenic zones in intractable epilepsy. Eur J Nucl Med Mol.

[54] Smith BJ, Karvelis KC, Cronan S, Porter W, Smith L, Pantelic MV (1999). Developing an effective program to complete ictal SPECT in the epilepsy monitoring unit. Epilepsy Res.

[55] Yassin A, Al-Mistarehi AH, El-Salem K, Urban A, Plummer C, Mohammadi S (2021). Effect of automatic injectors on the injection latency, safety, and seizure onset zone localization of ictal single photon emission computed tomography studies in adult epilepsy monitoring unit. Epilepsy Res.

