# Supplementary material for: Short post-injection seizure duration is associated with reduced power of ictal brain perfusion SPECT to lateralize the seizure onset zone
Source: EJNMMI Res. 2024 Apr 17;14:40. doi: 10.1186/s13550-024-01095-5 (PMC11024078; doi:10.1186/s13550-024-01095-5)
Supplement: Supplementary file 1 — Additional file 1. Criteria for the visual interpretation of ictal SPECT. [file 13550_2024_1095_MOESM1_ESM.pdf]

EJNMMI Research

Supplementary material

Short post-injection seizure duration is associated with reduced power of ictal brain perfusion SPECT to lateralize the seizure onset zone

Amir Karimzadeh[1,*], Kian Salimi[1,*], Berthold Voges[2], Ivayla Apostolova[1], Thomas Sauvigny[3], Michael Lanz[2], Susanne Klutmann[3], Stefan Stodieck[2], Philipp T. Meyer[4], Ralph Buchert[1] ORCiD ID 0000-0002-0945-0724

Departments of [1]Diagnostic and Interventional Radiology and Nuclear Medicine and [3]Neurosurgery, University Medical Center Hamburg-Eppendorf, Hamburg, Germany

[2]Department of Neurology and Epileptology, Protestant Hospital Alsterdorf, 22337 Hamburg, Germany

[4]Department of Nuclear Medicine, Medical Center - University of Freiburg, Faculty of Medicine, University of Freiburg, Freiburg, Germany

*These authors contributed equally as first authors

Criteria for the visual interpretation of ictal SPECT

- the SOZ is identified by regional hyperperfusion in cortical grey matter according to visual inspection of the uptake image and/or the statistical parametric map of ictal hyperperfusion
- the other way round is not true, that is, regional hyperperfusion in cortical grey matter is not automatically to be interpreted as the SOZ. For example, hyperperfusion in the insula and in the basal ganglia is often caused by seizure propagation from a SOZ in the ipsilateral temporal lobe. Hyperperfusion in the motor cortex and the supplementary motor area also might be secondary effects of the seizure and, therefore, might not indicate the SOZ
- there often is a "mirror focus" of the SOZ, that is, less pronounced hyperperfusion in the homologous brain region in the other hemisphere. In case of temporal seizures, the mirror focus often is restricted to the lateral part of the temporal cortex and is associated with less pronounced hyperperfusion in the insula, striatum, and thalamus in the hemisphere of the mirror focus. A mirror focus increases the certainty of the localitation of the SOZ within a specific brain lobe. However, it often reduces the certainty of the lateralization
- the SOZ might be identified in the uptake image only, that is, it is not required that the SOZ is confirmed by a cluster of significant hyperperfusion in the statistical parametric map (the rather conservative threshold of 3.0 z-score points is expected to result in some false negative findings). In particular, the SOZ might be identified by considerable left-right asymmetry in the uptake image. For example, a SOZ in the left temporal lobe might be identified by higher tracer uptake in the left temporal lobe compared to the right temporal lobe, although the (relative) increase of ictal tracer uptake in the left temporal lobe does not reach the level of statistical significance and, therefore, is not confirmed by a significant cluster in the statistical parametric map
- identification of the SOZ might also be primarily based on a cluster of significant hyperperfusion in the statistical parametric map, if on visual inspection of the uptake image the hyperperfusion does not appear very prominent (the conservative threshold of 3.0 z-score points is expected to result in a rather low rate of false positive findings)
- a statistically significant cluster of regional hyperperfusion located in white matter should not be interpreted as SOZ (it could be caused by mismatch of spatial resolution between the patient image and the normal database used for voxel-based testing, particularly when located close to the white-to-gray matter junction)
- a statistically significant cluster of regional hyperperfusion located in white matter can extend into cortical grey matter due to recovery effects caused by the limited spatial resolution of SPECT. This does not indicate hyperperfusion in cortical grey matter and, therefore, should not be interpreted as SOZ

- regional hyperperfusion in the cerebellum should not be interpreted as SOZ
- the ictal perfusion pattern in the cerebellum does not provide reliable information to support lateralization or localization of SOZ candidates in the cerebrum [1, 2]
- strongly pronounced regional hyperperfusion is usually associated with higher certainty of this region being the SOZ than mild hyperperfusion
- clear ictal hyperperfusion in the boundary of a (surgical) defect is associated with high certainty, because the actual hyperperfusion most likely is considerably underestimated due to partial volume effects
- the certainty that regional ictal hyperperfusion indicates the SOZ might be reduced if there is a defect or lesion in the homologous region in the contralateral hemisphere: the detected regional hyperperfusion might be a mirror focus due to propagation from the border zone of the defect/lesion while the hyperperfusion in the actual SOZ is not visible due to severe partial volume effects.
- a SOZ in the temporal lobe is supported (higher certainty) by hyperperfusion in the ipsilateral insula and/or striatum and/or thalamus and/or motor cortex (seizure propagation from the temporal lobe)
- a SOZ in the temporal lobe is supported (higher certainty) by hypoperfusion in the frontal lobe and/or parietal lobe and/or occipital lobe and/or precuneus (ictal surround inhibition). Regional hypoperfusion on ictal SPECT is often bilateral and does nor provide reliable information for the lateralization of the SOZ
- ranking of the certainty of the identification of the SOZ: visual identification of the SOZ in the uptake image <u>and</u> significant cluster of hyperperfusion in the statistical parametric map > visual identification in the uptake image only <u>or</u> significant cluster of hyperperfusion in the statistical parametric map only
- the score "no evidence of SOZ" in the first step is restricted to cases with (more or less) normal perfusion. The score "no evidence of SOZ" should not be used in cases with ≥ 2 SOZ candidates that all have about the same likelihood to be the actual SOZ. In the latter case, one SOZ candidate should be selected (with low certainty of lateralization and/or localization)
- in patients with structural lesions or perfusion defects, the visual read of the SPECT images should start with careful inspection of the boundary zone of the lesions/defects. After inspection of the boundary zone, the visual read should continue with the rest of the brain
- localization in the neighborhood of a structural lesion or a perfusion defect might increase the likelihood of a SOZ candidate to be the true SOZ. That is, if there are several candidates for the SOZ and one of these candidates is close to a structural lesion, this one might be preferred

- the visual read of the rest of the brain might start with the temporal lobes, since the temporal lobe is the pre-scan hypothesis for the localization of the SOZ in the majority (but not all!) of patients referred to perfusion SPECT for presurgical evaluation in most settings. However, the other brain lobes must not be neglected
- higher statistical significance of a regional cluster in the statistical hyperperfusion map might increase the likelihood of the cluster to correctly identify the actual SOZ. The statistical significance is indicated by the brightness of the color (the brighter the more significant).
- in cases with more than one SOZ candidate with similar significance in the statistical parametric map, the SOZ candidate in the temporal lobe (if there is one) might be preferred over the others (this rule is specific for the patient sample of the present study)
- the certainty of the localization of the SOZ in a specific brain lobe refers to the brain lobes in the hemisphere selected in the lateralization step (the brain lobes of the other hemisphere are not taken into account anymore at the localization step). This implies that the certainty of the localization within a brain lobe is independent of the certainty of the lateralization. The localization certainty can be high in case of low lateralization certainty (e.g., ictal hyperperfusion restricted to the temporal lobe in both hemispheres), and the localization certainty can be low in case of high lateralization certainty (e.g., ictal hyperperfusion that affects more than one lobe in one hemisphere). If there is only one SOZ candidate in the hemisphere selected at the lateralization step, the certainty of the localization is high, even if the SOZ is rather "weak" (only mild hyperperfusion in ictal SPECT). Thus, uncertainty associated with the "weakness" of a SOZ candidate should be mainly reflected in the certainty of the lateralization, not in the certainty of the localization.

References to the supplementary material

1. Dupont P, Zaknun JJ, Maes A, Tepmongkol S, Vasquez S, Bal CS, et al. Dynamic perfusion patterns in temporal lobe epilepsy. Eur J Nucl Med Mol Imaging. 2009;36:823-30. doi:10.1007/s00259-008-1040-6.

2. Shin WC, Hong SB, Tae WS, Seo DW, Kim SE. Ictal hyperperfusion of cerebellum and basal ganglia in temporal lobe epilepsy: SPECT subtraction with MRI coregistration. J Nucl Med. 2001;42:853-8.
